# Differing temperature dependencies of functional homologs zebrafish Abcb4 and human ABCB1

**DOI:** 10.3389/fphar.2024.1426040

**Published:** 2024-08-06

**Authors:** Till Luckenbach, Kathleen Burkhardt-Medicke

**Affiliations:** Department Ecotoxicology, Helmholtz Centre for Environmental Research—UFZ, Leipzig, Germany

**Keywords:** zebrafish Abcb4, human ABCB1, ATPase activity, stimulation, inhibition, temperature

## Abstract

The ATP binding cassette (ABC) transporters human ABCB1 and zebrafish (*Danio rerio*) Abcb4 are functionally homologous multixenobiotic/multidrug (MXR/MDR) efflux transporters that confer the efflux of a broad range of diverse chemical compounds from the cell. As ATPases, the transporters utilize the energy released by ATP cleavage for protein conformation changes and concomitant active transport of substrate compounds. The temperatures, at which human ABCB1 and zebrafish Abcb4 need to function, can substantially differ: Whereas the ambient temperature of human ABCB1, which is that of the human body, is constant, zebrafish Abcb4 has to be active in a wider temperature range as the body temperature of zebrafish can considerably vary, depending on the ambient water temperature (18°C–40°C). Here, we examined the effect of temperature on the ATPase activities of recombinant human ABCB1 and zebrafish Abcb4 generated with the baculovirus expression system. Incubation temperatures for enzyme reactions were set to 37°C and 27°C, corresponding to the human body temperature and the cultivation temperature of zebrafish in our lab, respectively. For stimulation and inhibition of zebrafish Abcb4 and human ABCB1 ATPase activities verapamil and cyclosporin A were added at different concentrations and 50% effect concentrations (EC50) were determined. The different temperatures had a stronger effect on the human ABCB1 than on the zebrafish Abcb4 ATPase: Differences between EC50 values for verapamil at 37°C and 27°C, respectively, were 1.8-fold for human ABCB1 but only 1.2-fold for zebrafish Abcb4. Activation energies (E_a_) of basal and verapamil-stimulated ATPases, calculated based on the Arrhenius equation, were 2-fold (basal) and 1.5-fold (verapamil-stimulated) higher for human ABCB1 than for zebrafish Abcb4. The differences between zebrafish Abcb4 and human ABCB1 ATPases in temperature sensitivity and activation energy could be important for the comparison of the functional properties of the two transporter proteins in the context of pharmaco-/toxicokinetics. Related to this, our finding that at equal reaction conditions the zebrafish Abcb4 ATPase activity tended to be generally higher than that of human ABCB1 may also be important, as this may point to a higher substrate compound transport rate of Abcb4.

## 1 Introduction

Certain members of the ATP-binding cassette (ABC) transporter protein family are cellular efflux pumps that with respect to their transported substrates are multispecific. Such transporter proteins are expressed in tissues with barrier functions (e.g., blood-brain barrier), conferring so-called multixenobiotic resistance (MXR) that is equivalent to multidrug resistance (MDR) of cancer cells in chemotherapy (Kurelec, 1992; Epel, 1998; Bard, 2000). The energy for protein conformation changes leading to the translocation of substrate compounds across cellular membranes comes from ATP cleavage. The action of ABC transporter proteins as efflux pumps is thus tightly connected to their activity as ATPases (Hamada and Tsuruo, 1988; Endicott and Ling, 1989).

The first described and most thoroughly studied human ABC transporter is ABCB1 or P-gylcoprotein (P-gp). It is an MDR transporter in cancer cells, which is expressed also in healthy cells in tissues with barrier functions, where it is an important player in pharmaco-/toxicokinetics (Gottesman and Ambudkar, 2001; Fromm, 2004).

Cellular ABCB1-like efflux activity appears to be highly conserved across all animal taxa, including fish (Luckenbach et al., 2014). In zebrafish, cellular MDR/MXR efflux activity is conferred by Abcb4, a functional homologue of human ABCB1 with a presumably similar range of transported substrates (Fischer et al., 2013; Fleming et al., 2013).

Due to its ease to be used for experiments and due to many similarities between humans and zebrafish the embryo of this species is an important model in pharmacology/toxicology (Scholz et al., 2008; Nishimura et al., 2015; Cassar et al., 2020; Patton et al., 2021).

In many aspects, however, zebrafish (embryos) and humans are not alike; morphological and physiological differences between the two systems can be a reason for differences in pharmaco-/toxicokinetics of chemicals leading to differences in internal concentrations that cause the effect. Thus, although the target associated with a chemical effect may be present in both systems it is essential to take specific structural features determining absorption, distribution, metabolism, and excretion (ADME) of a chemical in an organism into account when extrapolating experimental results obtained with zebrafish embryos to humans (MacRae and Peterson, 2015; Nishimura et al., 2015; Bauer et al., 2021).

Body temperatures of zebrafish and humans can considerably differ. Humans are endothermic organisms that maintain their body temperature constantly at around 37°C. The zebrafish, in contrast, is ectothermic, with its body temperature determined by that of the surrounding water. Zebrafish is a tropical fish, which was found to occur at water temperatures ranging from 18°C to 40°C in its natural environment (Spence et al., 2006; Engeszer et al., 2007; Arunachalam et al., 2013) and in culture is kept at a water temperature of around 24°C–29°C (Aleström et al., 2020).

Temperature is a strong determinant of enzyme activity and enzymes are adapted for optimal function at their natural ambient temperature range (Hochachka and Somero, 1968; Somero, 2004; Fields et al., 2015). Here, we addressed the question if functions of zebrafish Abcb4 and human ABCB1 are differently influenced by temperature. Abcb4 or ABCB1 ATPase activities were measured at different temperatures, with ATPase stimulating or inhibiting compounds added to the reactions. Recombinant zebrafish Abcb4 and human ABCB1 proteins obtained with the baculovirus-expression system were used in the experiments. The ATPase activities of the recombinant proteins were determined at 27°C, the temperature, at which zebrafish is kept in our lab, and 37°C, the human body temperature. Concentration series of verapamil and cyclosporin A, shown to stimulate and/or inhibit Abcb4/ABCB1 ATPase activities (Sarkadi et al., 1992; Sharom et al., 1995; Fischer et al., 2013), were added at different concentrations to the reactions. The concentration-effect data served as indicators for the sensitivities of the proteins for stimulation/inhibition at the different experimental temperatures. Based on the determined ATPase activities at the different temperatures, enzyme activation energies (E_a_) of the two transporters were calculated using the Arrhenius equation (Arrhenius, 1889).

## 2 Materials and methods

### 2.1 ABCB1 and Abcb4 ATPase assays

Protocols for production of recombinant zebrafish Abcb4 and human ABCB1 and performance of ATPase assays were adapted from Sarkadi et al. (1992) and Germann (1998) and are described in detail in Burkhardt-Medicke (2018). Briefly, recombinant zebrafish Abcb4 [NCBI Gene ID: JQ014001 (Fischer et al., 2013)] and human ABCB1 (NCBI Gene ID: 5243; obtained from ATCC, Manassas, VA, United States; ATCC number 65704) proteins were generated using the baculovirus expression system. ATPase assays were performed in 96-well plates enabling simultaneous incubations of treatments with various concentrations of the here applied transporter interacting compounds, verapamil and cyclosporin A, and controls. Verapamil and cyclosporin A stocks were set up in DMSO; DMSO was at 2% in experimental and control reactions. ATPase assays were run with concentration series of verapamil (0.6–120 µM) and cyclosporin A (0.001–40 µM). Each concentration was tested in one to five separate replication experiments. Care was taken that test compounds were dissolved at all applied concentrations in the experimental solutions by visual inspection for precipitate. Controls comprised negative controls (“basal transporter ATPase activity,” bA) and positive controls with 40 or 50 µm verapamil for maximum stimulation of transporter ATPase activities [“stimulated transporter ATPase activity,” sA; verapamil at both 40 μM and 50 µM caused maximum stimulation of the transporter ATPase activity (Bieczynski et al., 2021)]. Experiments comprised tests for concentration-dependent stimulation of the basal and inhibition of the verapamil-stimulated (40 µM or 50 µM) Abcb4/ABCB1 ATPase activities. Tests with recombinant Abcb4 or ABCB1 were run in 40 µL reactions in a water bath at 27°C for 40 min (Abcb4) or 20 min (ABCB1) or at 37°C for 20 min [for details on incubation times see Burkhardt-Medicke (2018)]. Reactions were stopped by adding 40 µL 5% SDS solution to each well. For colorimetric detection of free P_i_, 200 µL of detection reagent containing 8.33% ascorbic acid, 5.83 mM ammonium molybdate tetrahydrate and 2.5 mM zinc acetate were added to each well and absorption was measured at 750 nm.

### 2.2 Data analysis

#### 2.2.1 Concentration response relationships

Regressions describing concentration - ATPase activity relationships were calculated with the nonlinear four-parameter HILL model:

$$V\left(c_{t}\right)=bA+\frac{\left(mA-bA\right)}{1+{\left(\frac{{EC50}_{1/2}}{c_{t}}\right)}^{p}}$$ (1)

If data could not be fitted with the HILL model (Eq. 1) linear regression was used.

In addition, data for zebrafish Abcb4 ATPase stimulation by cyclosporin A were fitted using a modified Michaelis-Menten equation (Litman et al., 1997a; Litman et al., 1997b):

$$V\left(c_{t}\right)=\frac{{EC50}_{1\cdot }{EC50}_{2}\cdot bA+{EC50}_{2}\cdot mA{\cdot c}_{t}+iA\cdot {c_{t}}^{2}}{{EC50}_{1}\cdot {EC50}_{2}+{EC50}_{2}{\cdot c}_{t}+{c_{t}}^{2}}$$ (2)

In Eqs 1, 2 V(c_t_) is the Abcb4 or ABCB1 ATPase activity (in nmol Pi/min/mg membrane protein) at the concentration c_t_ of a test compound (t); bA is the basal ATPase activity (negative control); mA is the maximally stimulated ATPase activity (corresponds to sA in tests with verapamil, including the cyclosporin A inhibition test; in the cyclosporin A stimulation test mA corresponds to the mean value of maximum stimulation by cyclosporin A); iA is the ATPase activity at infinite c_t_ (Eq. 2); EC50 is the parameter value describing the c_t_ causing 50% of the maximum effect (EC50_1_: c_t_ causing half-maximum increment in ATPase activity; EC50_2_: c_t_ causing half-maximum reduction of ATPase activity from the sA value); p is the HILL number (Eq. 1).

Based on the regressions, EC50 values for stimulation of the basal (verapamil, cyclosporin A) and for inhibition of the verapamil-stimulated ATPase activities (cyclosporin A) were determined.

#### 2.2.2 Calculation of activation energy

The activation energy E_a_, which is the minimum energy required to start a chemical reaction, can be calculated based on the Arrhenius equation Eq. 3), describing the relationship of reaction rate k (i.e., enzyme activity) and temperature T (Arrhenius, 1889):

k=−A·eEaRT (3)

k is the reaction rate (enzyme activity in mol·L^−1^·s^−1^); A is the pre-exponential factor (number of collisions per second occurring with the proper orientation when all concentrations are 1 mol·L^−1^); R is the universal gas constant (8.314 J·K^−1^·mol^−1^); and T is the absolute temperature in K.

E_a_ was calculated using Eq. 4 based on the Arrhenius equation (Mortimer and Muller, 2003):

$$E_{a}=R\cdot \frac{T1\cdot T2}{T2-T1}\cdot \ln\left(\frac{k_{2}}{k_{1}}\right)$$ (4)

T1, T2 are different absolute temperatures and k1, k2 are the respective rate constants of the reaction.

Activation energies were calculated from the means of basal Abcb4/ABCB1 ATPase activities and of verapamil stimulated Abcb4 (40 μM, 120 μM) or ABCB1 (40 μM, 200 μM) ATPase activities.

All analyses were computed with GraphPad Prism 10 for macOS (GraphPad Software, San Diego, CA, United States).

## 3 Results

### 3.1 Abcb4 vs. ABCB1 ATPase activities

**FIGURE 1:**
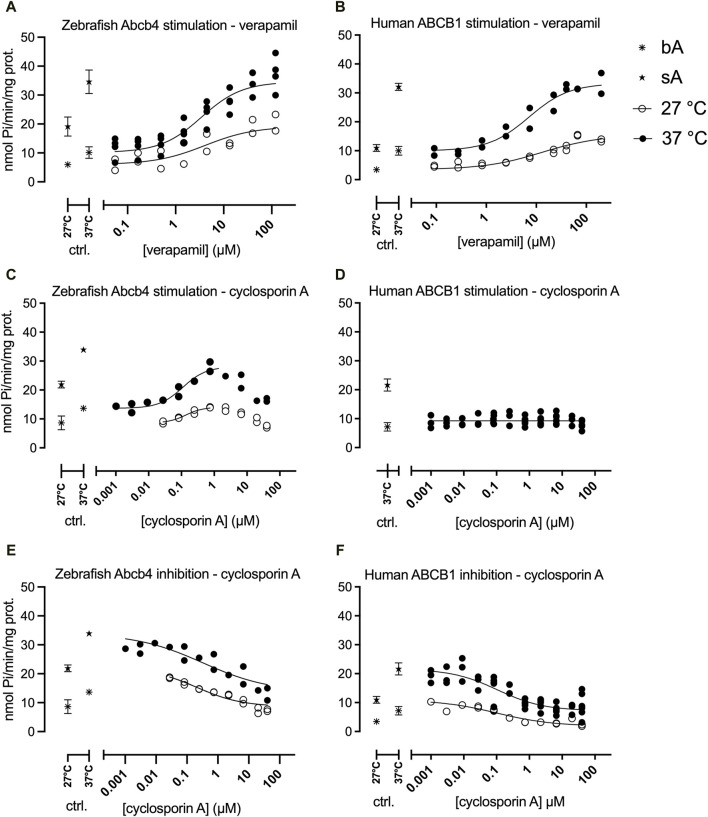
Zebrafish Abcb4 and human ABCB1 ATPase activities in the presence of different concentrations of verapamil (stimulation - **A, B**) or cyclosporin A (stimulation - **C, D**; inhibition - **E, F**). Stimulation of the basal or inhibition of the maximally verapamil-stimulated ATPase activities were measured. Regressions were calculated with the HILL model (Eq. 1), except for stimulation of the human ABCB1 ATPase by cyclosporin A (D; only at 37°C), which was fitted with linear regression. Data from each experimental replicate (n = 1–5 per tested concentration) are shown. Control (ctr., bA and sA at 27°C, 37°C) data are shown as means with standard deviations. If no error bar is depicted it is smaller than the symbol. bA: basal Abcb4 or ABCB1 ATPase activities; sA: maximally stimulated Abcb4 or ABCB1 ATPase activities (40 µM or 50 µM verapamil); mA_37°C_ = 28.1, mA_27°C_ = 14.0 (mA: maximum stimulation of zebrafish Abcb4 by cyclosporin A).

Zebrafish Abcb4 ATPase activities were generally higher than those of human ABCB1. Thus, over all experiments, the means of the bA and sA values for the tests at 37°C were 1.6- (bA) and 1.4-fold (sA) and for the tests at 27°C 2.2- (bA) and 1.9-fold (bA) higher for zebrafish Abcb4 than for human ABCB1 (see also Figure 1 for depiction of the bA and sA values in the different experiments).

### 3.2 Effects of test chemicals on Abcb4 and ABCB1 ATPase activities

Both zebrafish Abcb4 and human ABCB1 ATPase activities were stimulated by verapamil in a concentration-dependent way and the stimulated ATPase activities of both proteins were inhibited by cyclosporin A (Figure 1).

The maximum effect amplitudes (sA/bA quotient) at the different temperatures were similar for zebrafish Abcb4 (sA/bA_(27°C)_ = 3.2, sA/bA_(37°C)_ = 3.4) but by 25% different for human ABCB1 (sA/bA_(27°C)_ = 3.3, sA/bA_(37°C)_ = 4.4; Figure 1).

Effect concentrations (EC50) of verapamil (stimulation of bA) and of cyclosporin A (inhibition of sA) were in comparison lower for zebrafish Abcb4 than for human ABCB1 (Figure 1; Table 1). The differences between EC50 values for zebrafish Abcb4 and human ABCB1 were 2.1- (37°C) and 3.2- fold (27°C) for verapamil stimulation and 3.1- (37°C) and 4.2-fold (27°C) for cyclosporin A inhibition.

**TABLE 1:** Effect concentrations (EC50) of verapamil and cyclosporin A on zebrafish Abcb4 and human ABCB1 ATPase activities. EC50 values for stimulation of basal and inhibition of verapamil-stimulated ATPase activities at 27°C and at 37°C are given. Fold differences of the EC50 values at the two temperatures are also shown. EC50 values were calculated with the HILL model (Eq. 1; see also Figure 1).

	Compound	T (°C)	Zebrafish Abcb4		Human ABCB1	
			EC50 (µM) *(95% C.I.)*	$\frac{{\text{EC}50}_{27{}^{\circ}C}}{{\text{EC}50}_{37{}^{\circ}C}}$	EC50 (µM) *(95% C.I.)*	$\frac{{\text{EC}50}_{27{}^{\circ}C}}{{\text{EC}50}_{37{}^{\circ}C}}$
Stimulation	Verapamil	27	4.1 *(1.4–11.2)*	1.2	13.0 *(8.2–20.4)*	1.8
		37	3.5 *(2.2–5.3)*		7.2 *(4.9–10.5)*	
	Cyclosporin A	27	0.14 *(0.10–0.19)*	1.3	*No effect*	
		37	0.11 *(0.07–0.18)*			
Inhibition	Cyclosporin A	27	0.21 *(0.12–0.37)*	0.7	0.05 *(0.02–0.11)*	0.5
		37	0.32 *(0.10–0.96)*		0.11 *(0.05–0.20)*	

Stimulation of ATPase activity by cyclosporin A was only seen for zebrafish Abcb4 but not for human ABCB1 (Figure 1). Curve progressions of cyclosporin A concentration-dependent zebrafish Abcb4 ATPase activities were bell-shaped. At both temperatures (37°C, 27°C), Abcb4 ATPase activity was increasingly stimulated by rising cyclosporin A concentrations of up to about 1 μM, followed by decreasing Abcb4 ATPase activity (Figure 1). Modeling of the data with the Michaelis-Menten equation modified for enzyme kinetics with a bell-shaped progression (Eq. 2) was not possible; however, for the cyclosporin A concentration range with ascending Abcb4 ATPase activities HILL regression curves (Eq. 1) could be fitted. The EC50 value at 37°C was slightly lower than that at 27°C, showing a fold difference that was similar to that for verapamil at 37°C and 27°C, respectively, for the zebrafish Abcb4 ATPase activity (Table 1).

### 3.3 Effects of temperature on Abcb4 and ABCB1 ATPase activities

Both zebrafish Abcb4 and human ABCB1 ATPase activities were higher at 37°C than at 27°C. For zebrafish Abcb4, the means of all bA and all sA values at 37°C were 1.6-fold higher than the values for bA and sA, respectively, at 27°C. Means over all bA and sA values for human ABCB1 at 37°C were for both bA and sA 2.3-fold increased vs. the respective mean bA and sA values at 27°C (Figure 1).

**FIGURE 2:**
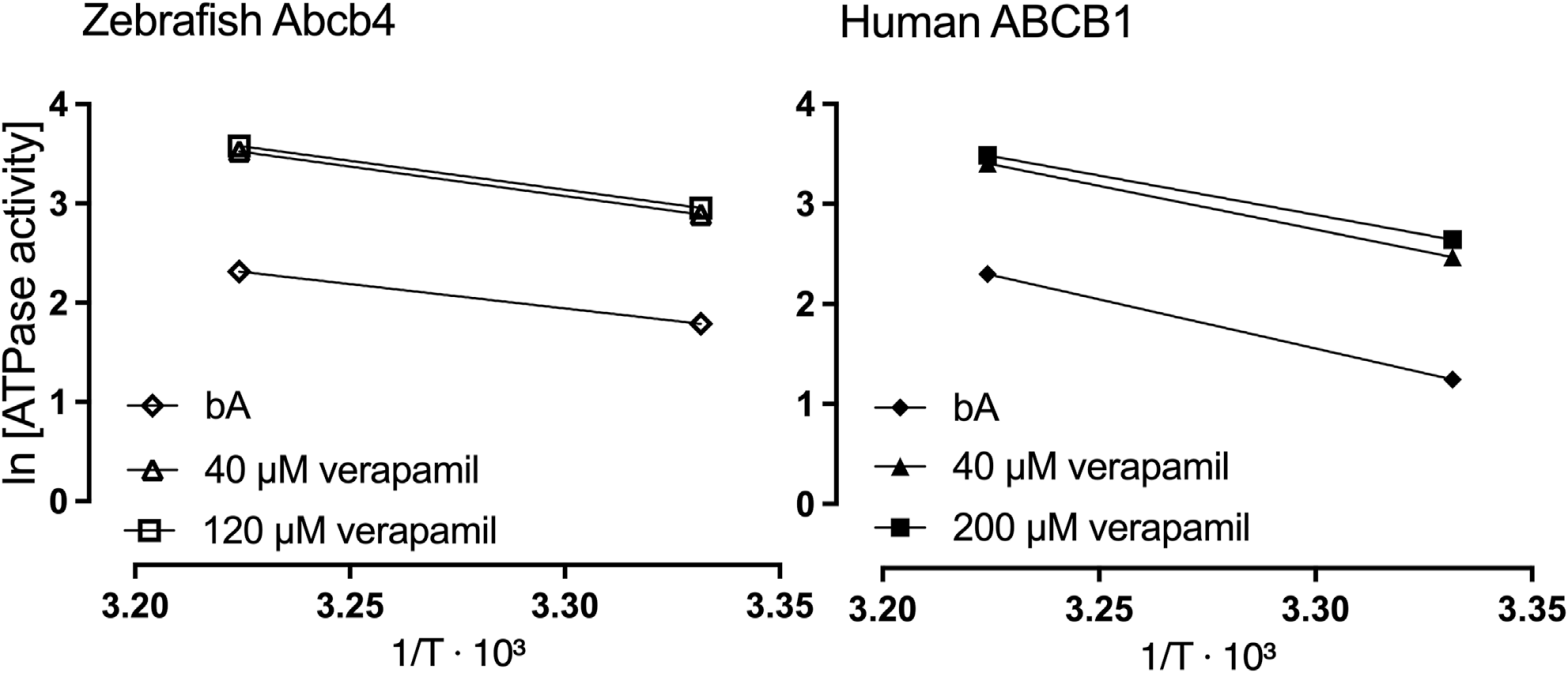
Arrhenius plots for Abcb4 and ABCB1 ATPase activities at 27°C (≙ 300.15 K) and 37°C (≙ 310.15 K). Depicted are ln of the means of ATPase activity data (Figure 1) for the bA control and tests with verapamil at the given concentrations.

The slopes of the linear regressions in the Arrhenius plots indicated a lower dependence on temperature of zebrafish Abcb4 than of human ABCB1. Whereas the slope of the linear regression for bA was m = −9.8 for ABCB1, it was m = −4.9 for Abcb4 (Figure 2). With verapamil added to the reactions, slopes of the linear regressions were m = −8.722 (40 µM) and m = −7.9 (200 µM) for human ABCB1 and m = −5.9 (40 µM) and b = −5.8 (120 µM) for zebrafish Abcb4 (Figure 2).

### 3.4 Abcb4 and ABCB1 ATPase activation energies

The ATPase activation energies of zebrafish Abcb4 and human ABCB1 varied up to 30% in the different reaction mixtures without and with verapamil or cyclosporin A (Table 2). Compared to the zebrafish Abcb4 ATPase, the activation energy of the human ABCB1 ATPase was 2- (bA) and 1.5- fold (40 µM verapamil) higher (Table 2). Activation energies calculated from Eq. 4 and from slopes (m; m = - E_a_/R) of Arrhenius plots (Figure 2), respectively, were almost identical (data not shown).

**TABLE 2:** Activation energies (E_a_; kJ/mol) of the zebrafish Abcb4 and the human ABCB1 ATPases. Depicted are E_a_ values for bA and for reactions with transporter ATPase stimulating agents present. Those were verapamil (Abcb4 and ABCB1) and cyclosporin A (Abcb4) at the given concentrations. E_a_ values were calculated with Eq. 4 (see also Figure 2). Data for verapamil were obtained from one single membrane preparation containing either recombinant Abcb4 or ABCB1; thus, potential variation across different membrane preparations could be avoided. Data for ATPase stimulation by cyclosporin A were obtained in assays with different Abcb4 membrane preparations.

		bA	ATPase stimulation				
			Verapamil			Cyclosporin A	
			40 µM	120 µM	200 μM	0.08 μM	0.74 μM
E_a_ (kJ/mol)	Abcb4	40.8	49.3	48.4		48.1	53.6
	ABCB1	81.6	72.4		65.2		

## 4 Discussion

The results obtained here clearly indicated differences in functional performance between zebrafish Abcb4 and human ABCB1. Using ATPase assays with recombinant protein we found 1) that ATPase activities of Abcb4 are higher than those of ABCB1 (see Section 3.1; Figure 1); 2) that the Abcb4 ATPase is less temperature-sensitive than the ABCB1 ATPase (Table 1); and 3) that the ATPase activation energy for Abcb4 is considerably lower than that for ABCB1 (Table 2).

Further differences between zebrafish Abcb4 and human ABCB1 were related to the ATPase activity responses of both proteins to transporter ATPase stimulating and inhibiting agents: Based on effect concentrations, zebrafish Abcb4 turned out to be more sensitive than human ABCB1 in the stimulation test with verapamil but less sensitive in the inhibition test with cyclosporin A than human ABCB1 (Table 1).

The effect concentration for verapamil for stimulation of the human ABCB1 ATPase determined here was in a similar range as found by others [EC50: 7.2 µM (at 37°C, Table 1); 4.8 µM (von Richter et al., 2009); 9.0 µM (Feng et al., 2008)].

Zebrafish Abcb4 ATPase was stimulated by cyclosporin A, as also reported earlier (Fischer et al., 2013), which was not the case with human ABCB1 (Figure 1). No or only slight ATPase stimulation of human/mammalian ABCB1 by cyclosporin A was also reported by others (Litman et al., 1997b; Sharom et al., 1995; von Richter et al., 2009). Cyclosporin A is a substrate of ABCB1, which raises the question how its translocation by the transporter protein can be performed without an input of additional energy by ATPase cleavage. It was suggested that the basal ABCB1 ATPase activity may be sufficient to transport cyclosporin A and requires no or only little additional energy, retained from weak ATPase stimulation, for transporter activity (von Richter et al., 2009). Basal ATPase activity was described as an intrinsic mechanistic property of ABCB1 (Al-Shawi et al., 2003) but there also is evidence that basal ATPase activity is caused by endogenous compounds, such as certain membrane lipids or hormones (Sharom, 2014; Stein and Litman, 2014; Tran et al., 2023); it thus is conceivable that the translocation of a non-ATPase-stimulating ABCB1 substrate, such as cyclosporin A, across the membrane by the transporter is via co-transport.

It may seem surprising that the zebrafish Abcb4 but not the human ABCB1 ATPase activity was stimulated by cyclosporin A. The bA level that was higher for zebrafish Abcb4 than for human ABCB1 could indicate that the energy turnover rate of the bA was higher in Abcb4 than in ABCB1. Stimulation of the zebrafish Abcb4 ATPase activity by cyclosporin A will provide even more energy for the transport of this compound by this transporter. Thus, the observation of stimulation of the zebrafish Abcb4 but not of the human ABCB1 ATPase activity by cyclosporin A may indicate that the transport of the compound by Abcb4 requires more energy than transport by ABCB1. However, it is also conceivable that a reason for non-existent stimulation of the ABCB1 ATPase activity by cyclosporin A is related to the higher activation energy of the ABCB1 ATPase: A consequence could be a higher ATPase stimulation threshold; thus, more compounds may be transported by ABCB1 without ATPase stimulation than by zebrafish Abcb4; zebrafish Abcb4 ATPase stimulation thus may be a more sensitive indicator of compounds with substrate properties. This could be further studied by performing measurements of transport rates of cyclosporin A and other non-ATPase-stimulating substrate compounds by zebrafish Abcb4 and human ABCB1 overexpressed in transfected cells using transwell systems, such as used by Kotze et al. (2024).

Both zebrafish Abcb4 and human ABCB1 showed higher ATPase activities at the higher experimental temperature. Besides a temperature effect on the ATPase enzymatic activity, it is also conceivable that the increased enzymatic activity at the higher temperature is related to differences in temperature-related membrane fluidity. ABC transporters are embedded in the cellular membrane and function and activity depend on the membrane environment [reviewed in Sharom (1997); Sharom (2014)] and earlier work demonstrated the influence of membrane constituents on the basal and stimulated ATPase activation energy of ABCB1 (Romsicki and Sharom, 1998; Al-Shawi et al., 2003). For the ATPase assays performed here membrane preparations containing the recombinant transporter proteins are used; the recombinant proteins thus are also embedded in cellular membranes. Evidence for the effects of temperature-related membrane fluidity on the enzymatic activity of membrane-based proteins has been obtained (Hazel, 1995). It seems conceivable that the membranes of Sf9 insect cells, cultivated at 27°C (Germann, 1998) and with the membrane fluidity adapted to this temperature, could be more brittle at 37°C, providing less support of the conformation of the embedded membrane proteins. If increased membrane fluidity had occurred at the higher experimental temperature (37°C), resulting in less support of the Abcb4/ABCB1 protein structure, greater variability of the ATPase activity data may have been expected. However, there was no clear trend of generally greater data variability in the 37°C than in the 27°C experiments (Figure 1). For instance, for bA and sA in the verapamil experiment with zebrafish Abcb4 (Figure 1A) percentages of the standard deviations from the means were 2.6% / 49.2% for bA / sA at 27°C and 19.5% / 11.7% for bA / sA at 37°C. Furthermore, the different effect amplitudes of the temperature differences on ATPase activities of zebrafish Abcb4 and human ABCB1 (Table 1) provide evidence that the different experimental temperatures affected ATPase enzyme activities of the two transporter proteins directly and not via temperature-related changes in membrane fluidity.

The temperature-related functional differences we found here for functionally homologous transporter proteins of an ectothermic (zebrafish) and endothermic vertebrate species (human) resemble those of a study comparing the temperature-dependent enzyme activities of succinic dehydrogenase (SDH) of frog (ectothermic) and rat (endothermic) (Vroman and Brown, 1963). As found here, the activation energy E_a_ of the SDH of the ectothermic was lower than that of the endothermic species; furthermore, the frog SDH enzyme activity changed with changing temperature to a lower degree than that of the rat SDH enzyme activity (Vroman and Brown, 1963).

Activation energy and temperature-sensitivity of the enzymes of the ectotherms that both were decreased compared to those of the enzymes of endotherms may indicate adaptation of enzymes to function at lower temperatures and a wider temperature range.

Resemblances of protein features, such as size and domain structure, and of functional properties indicate that the protein structures of the ABC transporters zebrafish Abcb4 and human ABCB1 are overall highly similar. This can be considered as remarkable as both proteins are dissimilar in about 1/3 of their amino acid sequences [64% amino acid sequence identity of Abcb4 and ABCB1; Fischer et al. (2013)], indicating that the according functional and structural properties of the proteins are determined by a comparatively small number of key amino acids in the sequence. The majority of similarities in amino acid sequences of the two proteins can be ascribed to the nucleotide binding domains (NBDs), which across taxa are highly conserved (Dean and Annilo, 2005); in contrast, transmembrane domain (TMD) sequences are very diverse across taxa. In a study on toxicant ABC transporters in bivalves it was suggested that the sequence variability of TMDs across taxa could be due to adaptations of these domains to the specific structures and properties of the specific cellular membrane environments of the transporter proteins in the different taxa (Luckenbach and Epel, 2008). It thus may be promising to specifically investigate TMD regions for structure determining amino acid patterns in the functionally homologous ABC transporters from different species.

A feature of zebrafish Abcb4 could be a less rigid protein structure (e.g., due to a smaller number of hydrogen bonds maintaining the protein structure) than that of human ABCB1, which may explain the smaller effect of the two different experimental temperatures on the Abcb4 ATPase activity (Table 1) and the lower activation energy of the Abcb4 ATPase enzymatic activity (Table 2).

The here presented findings are important in the context of the use of zebrafish embryos in compound screenings in human drug development. In compound screenings, zebrafish embyros are generally exposed to test compounds *via* the surrounding water; to assess the effect of a test compound it is essential to take into account how much of the test compound is taken up by the embryo tissue. Pharmaco-/toxicokinetics in zebrafish embryos depend on various life-stage-specific features, such as existence of a chorion or opening of the mouth that need to be considered (Nishimura et al., 2015). In addition, the ADME of chemical compounds in the zebrafish embryo are determined by transporters and metabolic enzymes (Luckenbach et al., 2014; Brox et al., 2016; Bauer et al., 2021). As energetic cost of active efflux of a compound acting as substrate of human ABCB1 in form of ATP hydrolysis, a stoichiometry of one ATP per effluxed substrate molecule was determined (Seelig and Li-Blatter, 2023). When assuming that the energy demand for substrate transport is identical for zebrafish Abcb4 and human ABCB1 (however, see above discussion of Abcb4 ATPase stimulation by cyclosporin A) a higher intrinsic ATP hydrolysis turn-over rate of zebrafish Abcb4 than of human ABCB1 (Section 3.1; Figure 1) may mean that zebrafish Abcb4 transports its substrates with a higher rate than human ABCB1. It thus would need to be investigated to which degree the polyspecific multidrug transporters zebrafish Abcb4 and human ABCB1 are not just homologous on a qualitative but also on a quantitative level. This could be done using the above mentioned transfected cells and transwell systems. Thus obtained results would be important to understand to which degree results obtained with zebrafish Abcb4 can be extrapolated to human ABCB1.

## 5 Conclusion

We found that based on ATPase activities functional homologs zebrafish Abcb4 and human ABCB1 differ with regard to energy turnover and temperature-related enzymatic activity. This likely reflects adaptation of respective enzyme activities to different temperature conditions. It could point to potential differences of the transporters regarding transport kinetics, with potential consequences for their roles on toxico-/pharmacokinetics. This could be further studied in the future using transport assays with transfected cell lines.

## Data Availability

The original contributions presented in the study are included in the article, further inquiries can be directed to the corresponding authors.
